# Licensed Nurse/Certified Nurse Aide Collaboration in the Care of Nursing Home Residents

**DOI:** 10.1093/geroni/igaa057.3322

**Published:** 2020-12-16

**Authors:** Cynthia Beynon

**Affiliations:** University of Utah, Bountiful, Utah, United States

## Abstract

This mixed-method study explores how nursing team collaboration is perceived and experienced in four nursing homes (NHs) in the Western United States. Licensed nurse (LN) and certified nurse aide (CNA) participants completed two survey tools to assess their perception of collaboration and teamwork in their current work environment. The LNs and CNAs were paired and interviewed both individually and as a caregiving pair to explore the lived experience of collaboration in the care of NH residents. Quantitative survey results were analyzed using IBM® SPSS Version 25, and participants reported a collaborative working environment with equally strong ratings in the following categories: partnership, cooperation, and coordination; they agreed with statements reflective of teamwork including team structure, leadership, situation monitoring, mutual support, and communication. No significant difference was found between LN and CNA responses or between team members in any of the four participating facilities. Qualitative survey data were loaded in NVivo12 and analyzed using a thematic analysis approach. The findings revealed five primary themes, including essential elements in successful team collaboration—perspective, coworker connection, communication, and mutual support—and ways teamwork and collaboration impact resident care.

